# Supplementary material for: Earnings management and readability of CSR report: Evidence from China
Source: PLoS One. 2024 Apr 3;19(4):e0301187. doi: 10.1371/journal.pone.0301187 (PMC10990201; doi:10.1371/journal.pone.0301187)
Supplement: S1 Appendix — (PDF) [file pone.0301187.s001.pdf]

Appendix: Variable definitions

Variables	sign	Definition
**Readability_CSR**		The average character and vocabulary of a single sentence, and the total pagesof CSR reports are homogenized and standardized to add for obtaining a readability index.
**Readability_AR**		We use character divided by average character of all the annual reports to obtain standardized characters, then we take the reciprocal of standardized characters to homogenize for obtaining readability of annual reports.
**Readability_CSR**		We add homogenized and standardized average character of single sentence and total pages of CSR reports to obtain alternative metrics for readability.
**Readability_CSR**		We add homogenized and standardized average vocabulary of single sentence and total pages of CSR reports to obtain alternative metrics for readability.
**DA**		Accrued earnings management is calculated by the modified Jones model as Model (3)-(5).
**Rem**		Real earnings management is calculated by the modified Roychowdhury model.
**Lev**	+	Leverage ratio estimated as total liabilities divided by total assets, both measured at the end of the year.
**Size**	-	The natural logarithm of the total assets.
**Roe**	+/-	Return on equity is equal to the net profit over end-of-period equity.
**Top1**	+	The shareholding ratio of the largest shareholder.
**Growth**	-	The owner of the equity growth equal to the percentage change in owners' equity from the last year to the current year.
**SOE**	-	A firm is classified as an SOE (state-owned enterprise) if its ultimate controlling shareholder is a state government, and as a non-SOE otherwise.
**Cash**	+/-	We divided monetary funds by current liabilities.
**Dual**	-	A dummy variable that equals one if the chairperson and CEO are the same person, and zero otherwise
**Attestation**	+/-	If companies have independent third-party authentication for CSR reports, the value is 1; otherwise, it is 0.
**Aggressiveness**		(The company's net profit for the year - the company's net cash flow from operating activities for the year)/the company's total assets at the beginning of the year.
**Pollution**		A dummy variable that equals 1 if the firm belongs to the heavy pollution industries, and 0 otherwise.
**Disclosure**		The value is 1 if CSR report of listed company is disclosed, otherwise, it is 0.
**Irregularities**		When the company has irregularities, the value is 1, otherwise, it is 0.
**ISO14001**		The value is 1 when the company has passed the ISO14001 certification, otherwise, it is 0.
**Internal control index**		We choose the Internal control index of the DIB database. When the value is greater than the median of all the samples, the value is 1, otherwise, it is 0.
**Debt costs**		Following Tran (2021), we divide financial expenses by total liabilities.

**Variablesy**	**sign**	**Definition**
**Equity costs**		Following Easton (2004), we calculate the quotient of the difference between EPSt+1 and EPSt+2 and the current price of stock and we take square root of the value.
**CSR performance**		Social welfare expenditure divided by total number of shares.
