# Supplementary material for: Earnings management and readability of CSR report: Evidence from China
Source: PLoS One. 2024 Apr 3;19(4):e0301187. doi: 10.1371/journal.pone.0301187 (PMC10990201; doi:10.1371/journal.pone.0301187)
Supplement: Attachment — Submitted filename: Review 1.docx [file pone.0301187.s004.docx]

Manuscript ID: PONE-D-23-29697R1

***Earnings management and readability of CSR report: Evidence from China***

**Comments to the author**

I sincerely congratulate the author(s) for conducting research on such an important topic. However, the author(s) may improve some areas for better clarity. Here are some comments on this matter:

1. In the abstract section the policy recommendation part is missing, if possible, try to incorporate it.
2. Authors must segregate the introduction and theoretical background separately to make the readers to understand this article easily without any confusion.
