# Supplementary material for: Earnings management and readability of CSR report: Evidence from China
Source: PLoS One. 2024 Apr 3;19(4):e0301187. doi: 10.1371/journal.pone.0301187 (PMC10990201; doi:10.1371/journal.pone.0301187)
Supplement: Attachment — Submitted filename: Response to Reviewers.docx [file pone.0301187.s005.docx]

**Response to Referee 1 On**

Thank you for your comments. Responding to them has enabled us to significantly improve the paper. The remainder of this memo explains our response in detail. We first quote each of your comments and then provide our response.

**Comment 1:** In the abstract section the policy recommendation part is missing, if possible, try to incorporate it.

**Response:** Thank you very much for your constructive suggestions. In the abstract section, I add that: “To improve the quality of information disclosure of listed companies, the recommendations are as follows: First, the government should issue CSR reporting standards to reduce the manipulation of Chinese CSR reports. Second, Chinese CSR reports disclosed by listed companies must be audited by independent third parties to enhance the credibility of the information. Third, the company needs to strengthen its external and internal supervision to reduce the manipulation space for the readability of Chinese CSR reports.”

**Comment 2:** Authors must segregate the introduction and theoretical background separately to make the readers to understand this article easily without any confusion.

**Response:** This is a good comment and we appreciate your advice. In the introduction, I delete that: “theory of behavior consistency is that individuals exhibit certain similarities and stability in their behaviors and behavioral styles in different scenarios. Chinese annual reports and Chinese CSR reports are different ways of disclosing information. When managers increase profits through earnings management, they may adopt consistent measures to manipulate the readability of Chinese annual reports and the readability of Chinese CSR reports. Existing research has proved that when managers increase profits through earnings management, the readability of American annual reports and the readability of Chinese annual reports may be reduced . Based on consistency theory, when managers reduce the readability of Chinese annual reports, the readability of Chinese CSR reports may be decreased. Therefore, when managers increase profits through earnings management, the readability of Chinese CSR reports may be reduced. Based on principal-agent theory, managers have an information advantage and shareholders have an information disadvantage. Chinese CSR reports disclose not only the social responsibility of the companies, but also the business information. Therefore, Chinese CSR reports are becoming an important channel for shareholders to obtain information. To make the right investment decision, shareholders read Chinese CSR reports carefully. Meanwhile, Chinese CSR reports are voluntary in China, largely unregulated, and do not have a widely enforced reporting framework. The readability of Chinese CSR reports is easier to manipulate than Chinese annual reports by managers. When managers artificially increase profits through earnings management, they may reduce the readability of Chinese CSR reports. The purpose is to prevent shareholders from detecting earnings management and increase information asymmetry between managers and shareholders. Based on impression management theory, managers may manipulate the readability of Chinese CSR reports to improve investors’ impression of the company. If investors detect that managers increase profits through earnings management, the impression of the company is negatively affected. To prevent investors from detecting the behavior of earnings management through reading Chinese CSR reports, managers may reduce the readability of Chinese CSR reports. It can give investors a good impression of the company.”

**Comment 3:** One of the reviewers may not download data.

**Response:** I have made all data underlying in my manuscript fully available. ICPS database requires registering an account to download data.

Thank you again for your help and we welcome additional comments that you may have.
