# Supplementary material for: Earnings management and readability of CSR report: Evidence from China
Source: PLoS One. 2024 Apr 3;19(4):e0301187. doi: 10.1371/journal.pone.0301187 (PMC10990201; doi:10.1371/journal.pone.0301187)
Supplement: Attachment — Submitted filename: Reviewer 1.docx [file pone.0301187.s006.docx]

Manuscript ID: PONE-D-23-29697R2

***Earnings management and readability of CSR report: Evidence from China***

**Comments to the author**

I sincerely congratulate the author(s) for conducting research on such an important topic. However, the author(s) may improve some areas for better clarity. Here are some comments on this matter:

1. In conclusion part try to summarise your whole study in few sentences like introduction, methodology, findings.
2. Put the limitations and future research work at the end after suggestions part.
