# Supplementary material for: Earnings management and readability of CSR report: Evidence from China
Source: PLoS One. 2024 Apr 3;19(4):e0301187. doi: 10.1371/journal.pone.0301187 (PMC10990201; doi:10.1371/journal.pone.0301187)
Supplement: Attachment — Submitted filename: Response to Reviewers.docx [file pone.0301187.s007.docx]

**Response to Referee 1 On**

Thank you for your comments. Responding to them has enabled us to significantly improve the paper. The remainder of this memo explains our response in detail. We first quote each of your comments and then provide our response.

**Comment 1:** In conclusion part try to summarise your whole study in few sentences like introduction, methodology, findings.

**Response:** Thank you very much for your constructive suggestions. In conclusion part, we state that: “When managers increase profits through earnings management, they may reduce the readability of Chinese CSR reports. The negative effect is more significant when companies are not punished for violations, when the internal control index of companies is low, when companies lack ISO14001 certification and when companies do not have independent third-party authentication for Chinese CSR reports. Further study shows that when managers increase profits through earnings management, they may reduce the readability of Chinese CSR reports by decreasing the readability of Chinese annual reports. When the value of earnings management just exceeds zero, the readability of Chinese CSR reports decreases. When managers reduce the readability of Chinese CSR reports, financing costs are improved and environmental performance is decreased.”

**Comment 2:** Put the limitations and future research work at the end after suggestions part.

**Response:** This is a good comment and we appreciate your advice. I put the limitations and future research work at the end after suggestions part.
