# Supplementary material for: Earnings management and readability of CSR report: Evidence from China
Source: PLoS One. 2024 Apr 3;19(4):e0301187. doi: 10.1371/journal.pone.0301187 (PMC10990201; doi:10.1371/journal.pone.0301187)
Supplement: Attachment — Submitted filename: Reviewer Comments.docx [file pone.0301187.s002.docx]

Manuscript ID: PONE-D-23-29697

***Earnings management and readability of CSR report: Evidence from China***

**Comments to the author**

I sincerely congratulate the author(s) for conducting research on such an important topic. However, the author(s) may improve some areas for better clarity. Here are some comments on this matter:

1. In order to improve the quality of the paper, please rewrite the abstract section by methodology part (sample, research method, time period of the study, etc) in a better way.
2. Provide a concise and clear statement of the research objective in the introduction to help readers to understand the main focus of the study
3. The authors are required to provide a strong theoretical background below the introduction section of the study separately.
4. The author has included a few recent publications in his/ her literature review while shaping his/ her arguments in the manuscript. Therefore, citing more recent literature might help the author to strengthen the hypothesis development.
5. In the literature review section, the research gap is missing. So, provide a small paragraph just below the literature review.
6. The author needs to provide clarity behind the uses of control variable and its impact on the dependent variable of the study.
7. The findings of the paper have definitely added value to the existing literature in the domain of CSR.
8. The authors are asked to develop the conclusion section by including the unique contributions of the paper, theoretical and managerial implications, limitations of the research, and future research directions.
9. Please cite relevant references wherever necessary.
a) Pareek, R., & Sahu, T. N. (2021). How far the ownership structure is relevant for CSR performance? An empirical investigation. *Corporate Governance: The International Journal of Business in Society*, *22*(1), 128-147. <https://doi.org/10.1108/CG-10-2020-0461>
b) Ghosh, S., Pareek, R., & Sahu, T.N. (2022). Does board size matter for CSR performance? A GMM-based dynamic panel data approach. *International Journal of Business Excellence,* Vol. ahead-of-print No. ahead-of-print. https://doi.org/ 10.1504/ijbex.2022.10049821
c) Agarwala, N., Pareek, R., & Sahu, T. N. (2023). Do Firm Attributes Impact CSR Participation? Evidence From a Developing Economy. *International Journal of Emerging Markets*. Vol. ahead-of-print No. ahead-of-print. <https://doi.org/10.1108/IJOEM-05-2022-0876>.
d) Ghosh, S., Pareek, R., & Sahu, T.N. (2022). The Role of Corporate Governance and Company Specific Characteristics on Environmental Disclosure Practices in India. *NIMMS Management Review,* *30*(4), 64-89. <https://doi.org/10.53908/NMMR.300404>
e) [Pareek, R.](https://www.emerald.com/insight/search?q=Ritu%20Pareek), & [Sahu, T.N.](https://www.emerald.com/insight/search?q=Tarak%20Nath%20Sahu) (2023) The nonlinear effect of executive compensation on corporate social responsibility performance. [*Rajagiri Management Journal*](https://www.emerald.com/insight/publication/issn/0972-9968), Vol. ahead-of-print No. ahead-of-print. <https://doi.org/10.1108/RAMJ-06-2022-0094>
f) Almahrog, Y., Ali Aribi, Z., & Arun, T. (2018). Earnings management and corporate social responsibility: UK evidence. *Journal of Financial Reporting and Accounting*, *16*(2), 311-332.
g) Ehsan, S., Nurunnabi, M., Tahir, S., & Hashmi, M. H. (2020). Earnings management: A new paradigm of corporate social responsibility. *Business and Society Review*, *125*(3), 349-369.
